# NOX Inhibition Improves β-Adrenergic Stimulated Contractility and Intracellular Calcium Handling in the Aged Rat Heart

**DOI:** 10.3390/ijms19082404

**Published:** 2018-08-15

**Authors:** Álvaro Valdés, Adriana V. Treuer, Guillermo Barrios, Nikol Ponce, Roberto Fuentealba, Raul A. Dulce, Daniel R. González

**Affiliations:** 1 Departamento de Ciencias Básicas Biomédicas, Facultad Ciencias de la Salud, Universidad de Talca, Talca 3460000, Chile; alvaldes@utalca.cl (Á.V.); adrianatreuer@hotmail.com (A.V.T.); gbarriosb@alumnos.utalca.cl (G.B.); nponce@utalca.cl (N.P.); rfuentealbaleyton@gmail.com (R.F.); 2 Interdisciplinary Stem Cell Institute, Miller School of Medicine, University of Miami, Miami, FL 33136, USA; RDulce@med.miami.edu

**Keywords:** NADPH oxidases, apocynin, VAS2870, aged heart, cardiomyocytes, isoproterenol, TBARS

## Abstract

Cardiac aging is characterized by alterations in contractility and intracellular calcium ([Ca^2+^]*_i_*) homeostasis. It has been suggested that oxidative stress may be involved in this process. We and others have reported that in cardiomyopathies the NADPH oxidase (NOX)-derived superoxide is increased, with a negative impact on [Ca^2+^]*_i_* and contractility. We tested the hypothesis that in the aged heart, [Ca^2+^]*_i_* handling and contractility are disturbed by NOX-derived superoxide. For this we used adults (≈5 month-old) and aged (20–24 month-old) rats. Contractility was evaluated in isolated hearts, challenged with isoproterenol. To assess [Ca^2+^]*_i_*, isolated cardiac myocytes were field-stimulated and [Ca^2+^]*_i_* was monitored with fura-2. Cardiac concentration-response to isoproterenol was depressed in aged compared to adults hearts (*p* < 0.005), but was restored by NOX inhibitors apocynin and VAS2870. In isolated cardiomyocytes, apocynin increased the amplitude of [Ca^2+^]*_i_* in aged myocytes (*p* < 0.05). Time-50 [Ca^2+^]*_i_* decay was increased in aged myocytes (*p* < 0.05) and reduced towards normal by NOX inhibition. In addition, we found that myofilaments Ca^2+^ sensitivity was reduced in aged myocytes (*p* < 0.05), and was further reduced by apocynin. NOX2 expression along with NADPH oxidase activity was increased in aged hearts. Phospholamban phosphorylation (Ser16/Thr17) after isoproterenol treatment was reduced in aged hearts compared to adults and was restored by apocynin treatment (*p* < 0.05). In conclusion, β-adrenergic-induced contractility was depressed in aged hearts, and NOX inhibition restored back to normal. Moreover, altered Ca^2+^ handling in aged myocytes was also improved by NOX inhibition. These results suggest a NOX-dependent effect in aged myocytes at the level of Ca^2+^ handling proteins and myofilaments.

## 1. Introduction

The aged population is increasing worldwide. Cardiovascular diseases are the leading cause of death worldwide and this becomes even more pronounced in the elderly population. In the heart, aging increases the risk of ischemic disease, atrial fibrillation, and heart failure. Cardiac aging involves a progressive degeneration and decline in function in absence of major cardiovascular risks, which makes the heart more vulnerable to stress and contributes to increased cardiovascular mortality, and morbidity. Cardiac aging includes hypertrophy, fibrosis, and apoptosis of myocytes [1] and at the functional level, the loss of the cardiac reserve [2].

In the heart, the aging process is associated with an increase in the production of reactive oxygen species (ROS) [3]. Traditionally, the increase ROS observed in the aged heart has been attributed to mitochondria since ROS are a byproduct of the mitochondrial respiratory chain. It has been hypothesized that accumulation of these ROS are a cause of aging due to cumulative cellular damage they produce in time. Although this theory is attractive and there is experimental data in support of it, other sources of ROS cannot be discarded [4].

Recent reports suggest that the nicotinamide adenine dinucleotide phosphate (NADPH) oxidases may be relevant in cardiac aging [5,6]. NADPH oxidases are a family of enzymes that generate reactive oxygen species (O_2_^−^ and H_2_O_2_) through electron transfer from NADPH to molecular oxygen. Seven family members of these oxidases are known, each based on a distinct catalytic subunit (NOX 1–5 and dual oxidase 1–2). This oxidase is a multi-sub unit enzyme composed of a membrane-bound heterodimeric subunit (p22^phox^ and gp91^phox^) and several cytoplasmic regulatory sub units including Rac1, p47^phox^, and p67^phox^. NADPH oxidases are crucial regulators of redox signaling through tightly regulated production of low ROS levels in the vicinity of target proteins. In addition, NOX activity has been hypothesized to contribute to cardiovascular aging [7], since NOXs are implicated in remodeling of the aging heart [8].

Several key protein involved in excitation–contraction coupling can undergo redox-sensitive alterations in activity, which contribute to myocardial contractile dysfunction. ROS generated by up regulated NADPH oxidase modulate Ca^2+^ transients [9] and myofilament function via post translational modifications in the aged heart [10]. Altered [Ca^2+^]*_i_* regulation is directly responsible for the attenuated contractility and relaxation observed in aged myocardium, which correlated with impairment of both cell shortening and relengthening [11].

The aging heart is characterized by changes in the Ca^2+^ handling of cardiac myocytes [12]. Particularly, Ca^2+^ removal from the cytosol by reuptake into the sarcoplasmic reticulum (SR) is decelerated in aged cardiac myocytes, favoring the development of diastolic dysfunction [13]. As a consequence, left ventricular (LV) diastolic dysfunction is a common condition in the elderly and manifested as exercises intolerance, dyspnea and fatigue [14].

The SR Ca^2+^-ATPase (SERCA2a) and its inhibitory protein, phospholamban (PLB), are important regulators of SR Ca^2+^ reuptake. SERCA2a, which couples the hydrolysis of one ATP molecule to transport Ca^2+^ into the SR, remove Ca^2+^ from the cytosol to facilitate relaxation. PLB is a negative regulatory protein of SERCA2a [15,16]. Dephosphorylated PLB inhibits SERCA activity, whereas PLB phosphorylation, by Ca^2+^/calmodulin or cAMP dependent protein kinases, relieves this inhibition. Decreased SERCA2a pumping rate, mRNA and protein levels, and decreased phospholamban have been reported in senescent rat and human myocardium, while others have reported no change in mRNA and protein level of both SERCA2a and PLB with age [17].

Since we previously described a role for NOX in the dystrophic cardiomyopathy [18], the aim of the present study was to determinate the effect of NOX inhibition on the aged cardiomyocyte contractile function.

## 2. Results

### 2.1. General Characteristic of Adult and Senescent Rats

The baseline characteristics of the animals in the study and hemodynamic parameters are presented in Table 1. Body weight was increased in senescent compared to adult rats. Heart weight was also increased in aged rats, emphasizing that in the rat, aging is associated with cardiac hypertrophy. Nevertheless, aged hearts displayed diminished ventricular function evaluated as developed pressure, contractility (dP/dt_max_) and lusitropy (dP/dt_min_).

### 2.2. Isoproterenol Response in the Isolated Heart

**Figure 1:**
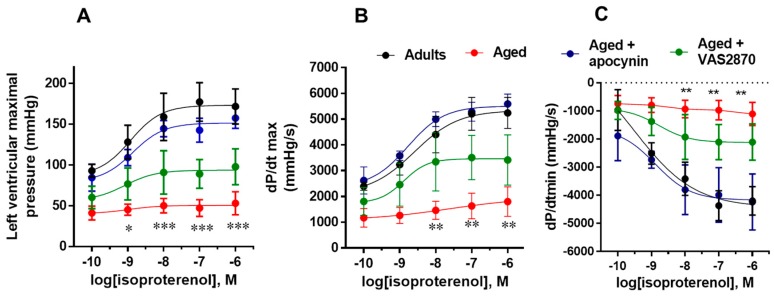
Heart function in the Langendorff preparation. Adult and aged isolated rat hearts were challenged with increasing concentrations of isoproterenol. Contractility was evaluated as developed pressure, dP/dt_max_ and lusitropy as dP/dt_min_. Aged hearts were treated with NOX inhibitors apocynin (100 µmol/L) or VAS2870 (20 µmol/L). *N* = 5 hearts each group. Values are presented as mean ± standard error; *n* = 5. * *p* < 0.05, ** *p* < 0.01 and *** *p* < 0.001 vs. corresponding control.

To assess cardiac ventricular function in the aged myocardium, we submitted isolated hearts from adult and aged rats to stimulation with increasing concentrations of isoproterenol, a β-adrenergic agonist. We observed that adult hearts displayed a positive inotropic and lusitropic response to increasing concentrations of isoproterenol, while aged hearts showed a significantly diminished response (Figure 1). Next, we treated aged hearts with NOX inhibitors. Apocynin (100 µM) produced a complete recovery of developed pressure, dP/dt_max_ and dP/dt_min_ in aged hearts. On the other hand, apocynin had no effect on adult hearts (supplementary material). A more specific NOX inhibitor, VAS2870 (20 µM) also produced a significant recovery of ventricular function in aged hearts, although not as complete as apocynin. These results suggest for a role of NOX in the diminished cardiac reserve in the aged rat heart.

### 2.3. Isolated Cardiomyocytes

Next, to gain more insights into the cellular mechanisms involved in the effect of the NOX inhibitors and to rule out the influence of vascular effects, we designed experiments in isolated cardiomyocytes.

Baseline characteristic of isolated myocytes are indicated in Table 2. Regarding sarcomere shortening, aged myocytes presented prolonged time 50 to peak shortening (TS_50_) and prolonged T_50_ to relengthening.

#### 2.3.1. Sarcomere Shortening

**Figure 2:**
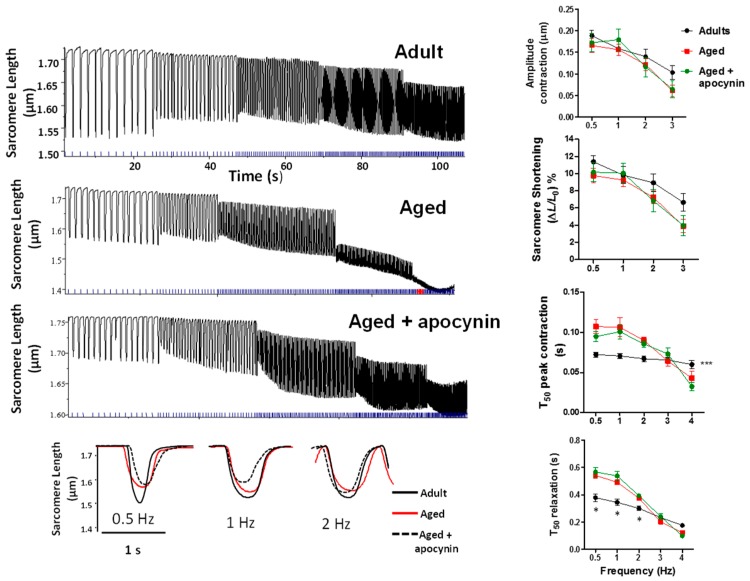
Contractility in isolated myocytes. Isolated cardiomyocytes stimulated at increasing frequencies. The left panels show representative traces of original trains of stimulation in adult (*n* = 19), aged (*n* = 22), and aged myocytes treated with apocynin 50 µmol/L (*n* = 16). The lower panel shows single twitch traces from each group (superimposed for comparison), of cardiomyocytes stimulated at 0.5, 1, and 2 Hz. The graphics to the right depict the analysis of sarcomere shortening, time to reach 50% of peak shortening (T_50_ peak contraction) and time to reach 50% (T_50_ relaxation). * *p* < 0.05 vs. aged and aged + apocyinin, *** *p* < 0.001 vs. aged and aged + apocyinin.

Isolated cardiomyocytes were submitted to a train of increasing frequency rates of stimulation, from 0.5 to 4 Hz (Figure 2). In adult myocytes, the amplitude of this response in sarcomere shortening is usually negative. This was the case for both adult and aged myocytes, although many aged myocytes could not complete the train of stimulation, not being able to reach 4 Hz. There were no differences in shortening amplitude between adult and aged myocytes and this amplitude was not further changed by apocynin treatment. The kinetic parameters T_50_ of peak shortening was increased in aged myocytes (*p* < 0.05), and was not improved by apocynin treatment. The same was obtained T_50_ of relaxation.

#### 2.3.2. Intracellular Ca^2+^ Transients

**Figure 3:**
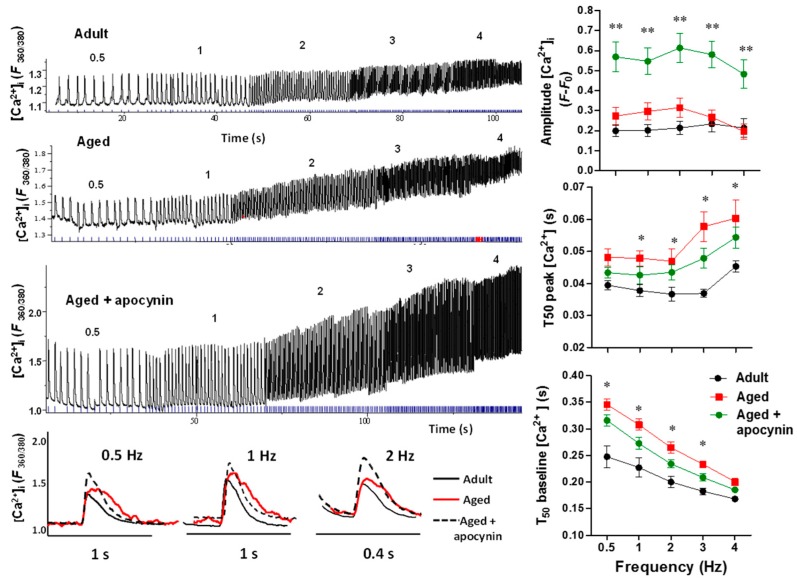
Intracellular Ca^2+^ transients. [Ca^2+^]*_i_* transients in response to increase in frequency of stimulation from 0.5 to 4 Hz. Upper left panel, representative traces of intracellular calcium in myocytes from adult (*n* = 17), aged (*n* = 23), and aged myocytes treated with apocynin 50 µmol/L (*n* = 18). The numbers on the traces indicate the frequency of stimulation. The lower panel shows representative traces with more temporal resolution depicting transients evoked at 0.5, 1, and 2 Hz. The graphs depict the amplitude of [Ca^2+^]*_i_* transients, time 50 to reach peak [Ca^2+^]*_i_* (T_50_ peak [Ca^2+^]*_i_*, and time 50 to reach relaxation (T_50_ baseline [Ca^2+^]*_i_*). * *p* < 0.05 vs. adult, ** *p* < 0.001 vs. adult.

In parallel to myocyte shortening, we evaluated intracellular Ca^2+^ in isolated myocytes (Figure 3).

[Ca^2+^]*_i_* transients amplitude was slightly increased in aged cardiomyocytes (*p* < 0.05) and were further increased by apocynin treatment. T_50_ to peak Ca^2+^ was increased in aged myocytes (*p* < 0.05), and was not improved by the apocynin treatment. T_50_ to peak relaxation was increased in aged myocytes (*p* < 0.05) and reduced towards normal by apocynin treatment. These results suggest impairments in the release and reuptake of cytosolic Ca^2+^ in aged myocytes.

#### 2.3.3. Myofilaments Ca^2+^ Sensitivity

**Figure 4:**
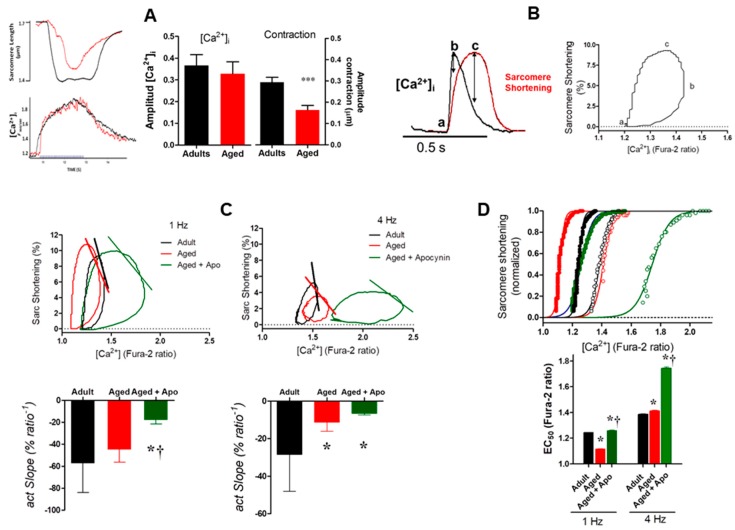
Myofilaments Sensitivity. (**A**) Using thapsigargin to block SERCA activity, myocytes were submitted to tetanic stimulation (40 Hz) to evaluate the myofilaments Ca^2+^ sensitivity. (Left panels): contraction (up) and the [Ca^2+^]*_i_* evoked by tetanic stimulation. (Right panels): pooled data for the amplitude of contraction (evaluated as degree of sarcomere shortening) and the amplitude of the cytosolic Ca^2+^ concentration evoked by tetanic stimulus in adult and aged cardiomyocytes. *** *p* = 0.0002. *N* = 20 cells from adult (*n* = 3) hearts and 29 cells from aged hearts (*n* = 3). (**B**) Phase loop analysis for the sarcomere shortening-[Ca^2+^]*_i_* relationship. Example of hysteresis analysis of Ca^2+^ vs. sarcomere shortening for a normal twitch. The segment from a-b represents the Ca^2+^ activating phase, the b–c segment represents cross-bridge feedback, and the a-c segment represents the relaxation phase. (**C**) Hysteresis loops used to obtain the slope of the activation phase at 1 and 4 Hz. (**D**) sarcomere shortening-[Ca^2+^]*_i_* in the activating phase fitted to the Hill equation to obtain the EC_50_, the Ca^2+^ concentration that activates the 50% of the maximal shortening (normalized). The filled symbols represent determinations obtained at 1 Hz of stimulation; the open symbols represent determinations obtained at 4 Hz of stimulation. *N* = 14 adults, 16 aged and 11 aged + apocynin cells for experiments at 1 Hz; 9 adult, 9 aged, and 7 aged + apocynin cell for experiments at 4 Hz. * *p* < 0.001 vs. adult. † *p* < 0.001 vs. aged.

The diminished degree of contraction associated with sustained Ca^2+^ transients in aged myocytes suggests reduced Ca^2+^ sensitivity in the aged heart cells. We tested this hypothesis using a protocol treating the myocytes with thapsigargin, a pharmacological SERCA blocker. After incubation with thapsigargin, myocytes were submitted to tetanic stimulation (40 Hz) to evaluate the myofilaments Ca^2+^ sensitivity (Figure 4A). While the amplitude of the level of [Ca^2+^]*_i_* evoked by stimulation was not different between adult and aged cardiomyocytes, the tetanic contraction was reduced in aged myocytes (*p* < 0.05), suggesting reduced Ca^2+^ sensitivity.

To further investigate the sensitivity to Ca^2+^ and myofilament interaction in these cardiomyocytes, we plotted sarcomere shortening vs. [Ca^2+^]*_i_* to obtain the hysteresis loops (Figure 4B). Slower relaxation is associated with sensitization of myofilaments to Ca^2+^ and increased affinity. Accordingly, we analyzed sarcomere shortening-[Ca^2+^] hysteresis loops of cardiomyocyte contractions induced by field-stimulation at either 1 or 4 Hz, in order to detect differences in responsiveness to Ca^2+^. In general, the morphology of the loops was greatly affected in cardiomyocytes from aged rats. We observed a general frequency-dependent myofilament desensitization to Ca^2+^ (shift to the right of the loops) from 1 to 4 Hz, which was more pronounced in the aged groups (either in the absence or presence of apocynin) compared to the adult group (Figure 4C). From the analysis of the relaxation phases (segment transitioning between the maximum and minimum sarcomere length shortening), it was observed a differential behavior depending on the stimulation rate (Figure 4C). At 1 Hz, the aged myocytes exhibited increased sensitivity compared to adult controls, however this difference was inverted by increasing frequency (4 Hz) (Figure 4D). This substantial reduction in myofilament sensitivity to Ca^2+^ in the aged group by increasing pacing is consistent with our results in tetanized cardiomyocytes shown in Figure 4A. The treatment of aged myocytes with apocynin induced a robust desensitizing effect at the studied frequencies (Figure 4C,D).

The relationship Ca^2+^-sarcomere shortening was further assessed through the analysis of the force activation phase of the sarcomere shortening-[Ca^2+^]_i_ loops (which is located between the maximum [Ca^2+^]_i_ and the maximum contraction as indicated in Figure 4C by lines), in order to evidence the effect of aging on the cross-bridge cycling. At 1 Hz, the slopes of the activation phase (actSlope) obtained by linear regression of the shortening-[Ca^2+^]_i_ pairs within the mentioned segment of the loop (Figure 4C, bar graphs), were virtually similar in both groups, adults and aged. However, the increasing stimulation induced a decrease in the force-activation slope in myocytes from the aged rats, which might indicate deteriorated cross-bridge cooperativity. Consistently, apocynin promoted a reduction in the force activation slope at 1 Hz and did not correct the impaired myofilament interaction at 4 Hz in aged cardiomyocytes. However this treatment was able to preserve the contractile performance in these cells by increasing the systolic [Ca^2+^]_i_ and consequently the [Ca^2+^]_i_ amplitude.

### 2.4. Cardiac Oxidative Stress

**Figure 5:**
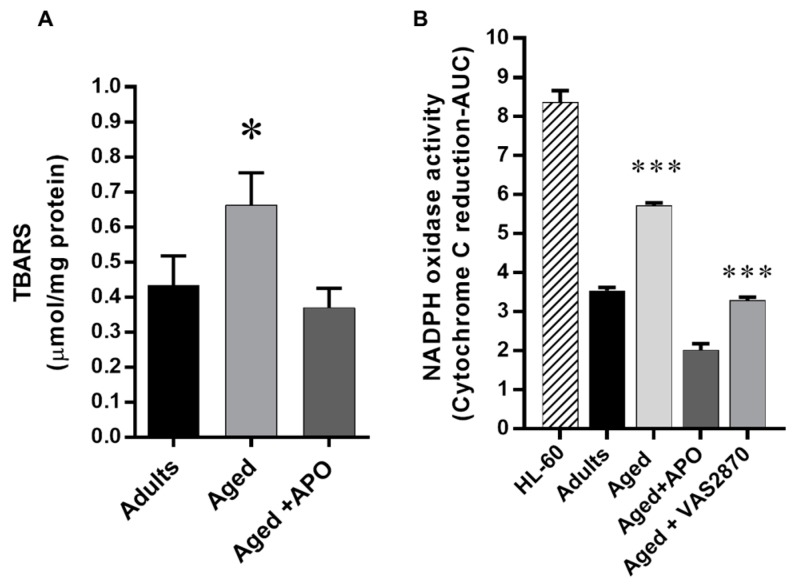
Oxidative Stress. NOX-derived oxidative stress was investigated by two methods. Upper panel, Western blot for p47phox, one of the activating subunits of NOX2, in adults (*n* = 4) and aged hearts (4) and the quantification normalized to GAPDH levels (mean ± standard deviation). (**A**) The graph depicts the concentration (mean ± SEM) of thiobarbituric acid (TBARS), a marker of lipid peroxidation, in cardiac homogenates from adult (*n* = 4), aged (*n* = 5), and aged hearts treated with apocynin (APO) 100 µmol/L (*n* = 5). (**B**) NADPH oxidase activity assessed as cytochrome c reduction. The graph depicts the reduction of cytochrome c, activity over time is shown as area under the curve (AUC) in cardiac homogenates from adults (*n* = 3), aged (*n* = 3), aged treated with apocynin 100 µmol/L (APO, *n* = 4), and aged hearts treated with VAS2870 (20 µmol/L, *n* = 4). * *p* < 0.05 vs. adult and aged plus apocynin, HL-60 cells were used as positive control. *** *p* < 0.05 vs. all the other groups.

NOX-derived oxidative stress was investigated by two methods (Figure 5). First, we evaluated the concentration of thiobarbituric acid (TBARS), a marker of lipid peroxidation in cardiac homogenates (Figure 5A). TBARS concentration was 0.43 ± 0.08 µmol TBARS/mg protein in adults hearts, was increased in aged hearts to 0.66 ± 0.09, and was reduced towards normal in the aged hearts that were treated with apocynin: 0.37 ± 0.05 µmol TBARS/mg protein (*p* < 0.05). This confirms that use of the NOX inhibitor reduced the cardiac oxidative stress. To verify the NADPH oxidase origin of this oxidative stress, we evaluated NADPH oxidase activity indirectly, by the cytochrome c reduction method (Figure 5B). Aged hearts showed increased NADPH oxidase activity compared to adults hearts. This activity was reduced in aged hearts that were treated both with apocynin (100 µmol/L) and VAS2870 (20 µmol/L): 3.54 ± 0.04 area under the curve units (absorbance × time) in adults hearts, 5.72 ± 0.04 in aged hearts, 2.02 ± 0.08 in aged hearts treated with apocynin and 3.28 ± 0.04 in aged hearts treated with VAS2870 (*p* < 0.001).

#### Expression of NOX Isoforms

**Figure 6:**
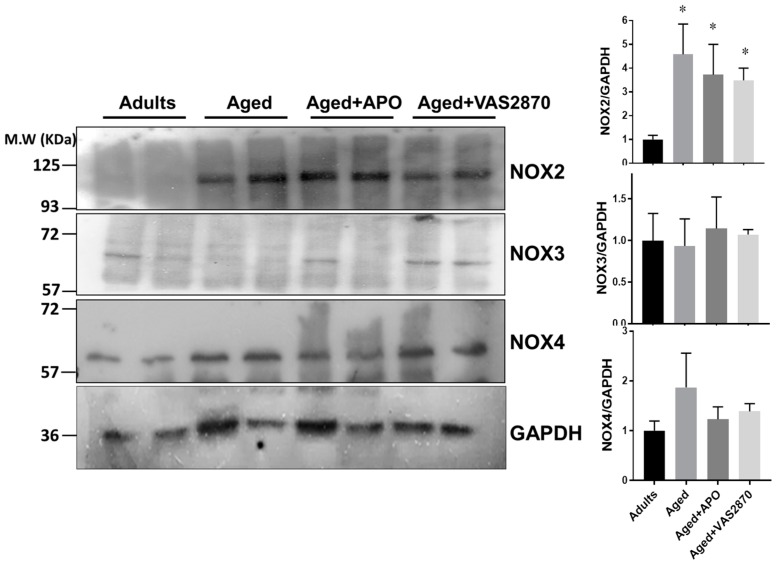
NOX isoforms expression. NOX isoforms expression was investigated by Western blotting. The left panels show representative Western blots of NOX isoforms (NOX2, NOX3, and NOX4) in cardiac homogenates from adults, aged, aged treated with apocynin 100 µmol/L (Aged + APO), and aged hearts treated with VAS2870, 20 µmol/L. GAPDH levels were used as loading control. The bar graphs depict average results normalized by GAPDH levels. *N* = 4 in each group. * *p* < 0.05 vs. adults.

To gain further insight into the origin of the superoxide source in aged hearts, we examined the levels of NOX isoforms of the different groups in our study. For this purpose we analyzed the levels of NOX2, NOX3, and NOX4 in cardiac homogenates by Western blotting (Figure 6). NOX2 was increased in aged hearts compared to adults and this remained elevated in the hearts that were treated with the NOX inhibitors (*p* < 0.05). In the case of NOX3, in our hands it was barely detected and was not different between groups. For the case for NOX4, no differences between groups were found. These results suggest that NOX2 is the origin of increased superoxide in the aged hearts.

### 2.5. Ca^2+^-Handling Proteins Levels

**Figure 7:**
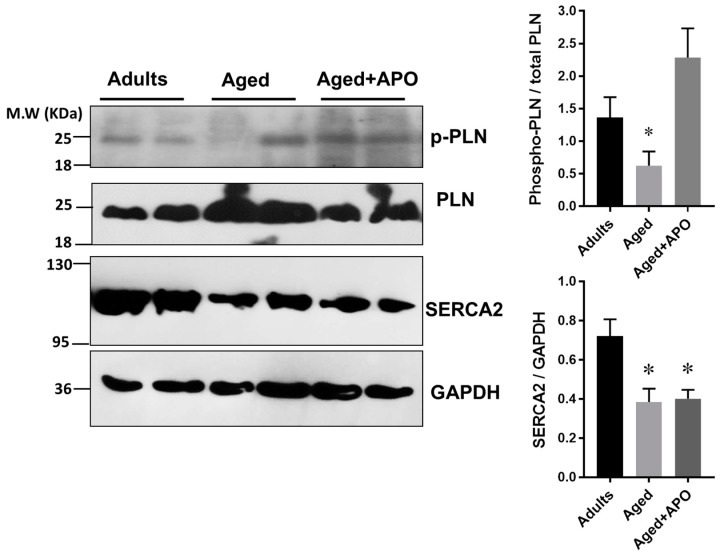
Calcium handling proteins. Left, representative images of Western blots for phospho-phospholamban pPLN (Ser16/Thr17), total phospholamban, and SERCA2 and, from adult, aged, and aged hearts treated with apocynin (APO, 100 µmol/L), all previously challenged with isoproterenol 100 µmol/L. The graphs to the right depict the densitometry analysis (mean ± standard deviation), normalized to GAPDH levels. *N* = 4 hearts in each group for phospholamban analysis. *N* = 4 adults and *n* = 6 in aged and aged + APO for SERCA analysis. * *p* < 0.05 vs. adults.

Finally, since the kinetic parameters of Ca^2+^ release and reuptake were altered in aged myocytes, we decided to look into the levels of Ca^2+^ handling proteins in cardiac homogenates (Figure 7). SERCA2 and phospholamban (PLN) expression and the degree of phospholamban phosphorylation in adults and aged hearts were assessed by Western blotting. First, we examined the levels of phosphorylated phospholamban (PLN) in both serine 16 and threonine 17 (the antibody recognizes PLN only when is phosphorylated at both sites) of hearts that were treated with 100 µmol/L isoproterenol. The degree of phosphorylation of pentameric PLN (molecular weight ≈ 25 kDa) was reduced in aged hearts compared to adults and was increased in aged hearts treated with apocynin evaluated as the ratio of phosphorylated to total phospholamban: 1.36 ± 0.31 in adults, 0.62 ± 0.21 in aged hearts, and 2.29 ± 0.44 in aged hearts treated with apocynin (*p* < 0.05). SERCA2 levels were reduced in aged hearts, and were not changed by apocynin treatment: 0.72 ± 0.08 in adults, 0.38 ± 0.07 in aged hearts, and 0.40 ± 0.045 ratio of SERCA/GAPDH levels in aged hearts treated with apocynin (*p* < 0.05). Consequently, the ratio of SERCA to PLB was decreased in aged hearts (*p* < 0.05). This suggests that in the aged rat myocardium, the capacity for Ca^2+^ reuptake is diminished by reduced levels of SERCA2, and upon adrenergic stimulation, PLN phosphorylation is impaired.

## 3. Discussion

In the aged myocardium, increased ROS are associated with a decrease in function, as well as on ventricular remodeling. This ROS production has been ascribed traditionally to the mitochondria.

In the heart, both NOX2 and NOX4 are expressed. NOX2 is located in the sarcolemma while NOX4 is located in the mitochondrial matrix [19]. It is possible that in the aged rat heart, both isoforms may have contributed to the increased oxidative stress, since apocynin completely recovered ventricular function in the isolated heart, while VAS2870, another NOX2 specific inhibitor restores partially this function.

Here we observed that the aged heart presents a diminished response to β-adrenergic stimulation compared to adult heart and for the first time show that acute NOX inhibition is able to restore this fundamental mechanism of cardiac inotropism. At the subcellular level, we observed that aged myocytes presented a slightly increase in amplitude of Ca^2+^ transients and reduced myofilaments Ca^2+^ sensitivity. Apocynin, a NOX inhibitor increased the amplitude of these transients. Our results suggest that this agent improved the function of the RyR2 and SERCA2, improving the Ca^2+^ cycling, although the molecular mechanism remains to be determined.

There is general agreement that in the aged myocyte, the activating L-type Ca^2+^ is reduced [20], the activity of the RyR2 is reduced (at the level of Ca^2+^ sparks) [21,22] and SR Ca^2+^ reuptake is slowed [23], in part by reduced SERCA expression, making Ca^2+^ transients smaller in amplitude and slower [13]. Several of these observations have been confirmed in the present work. NOX inhibition may have influenced both SERCA and RyR2, in a redox-sensitive manner, since all the pharmacological maneuvers performed in this study were acute. Particularly, RyR2 is extremely redox sensitive [24], due to its large number of cysteines residues [25,26].

Our results agree with those observed by Rueckschloss et al. [27], who observed similar effects of NOX inhibition in aged compared to young mouse myocytes. Nevertheless, in our model we evaluated in the intact heart the β-adrenergic response. Furthermore, contrary to Rueckschloss et al., we observed significant changes in the Ca^2+^ handling proteins SERCA and in the ratio SERCA/phospholamban.

These results suggest that NOX-derived superoxide may have produced oxidative modifications in Ca^2+^ handling and myofilaments proteins. Cooper et al. showed that in the aged rabbit heart, RyR2 is oxidized, although in that model the source of ROS was mitochondrial [28]. In the aged mouse, Andersson et al. [29] found increased S-nitrosylation of RyR1. On the other hand, Qin et al. found in the aged mouse heart that SERCA is oxidized by sulfonation at cysteine 674, decreasing its activity, although the source of ROS was not identified [30].

This increased activity in NOX activity in the aged heart has been proposed to depend on nitric oxide 1 (NOS1) [31] and on the renin-angiotensin system [8].

In isolated myocytes, apocynin produced an increase in the calcium transients, as well as a reduction in the myofilaments Ca^2+^ sensitivity. Although this effect did not altered relaxation parameters in isolated myocytes, probably due to the fact that these cells are unloaded, these effects may have improved relaxation and lusitropy, which are reduced in isolated aged hearts.

Indeed, the role of myofilaments in this model of aging is complex. In general, at higher frequencies of stimulation, Ca^2+^ sensitivity was reduced in aged myocytes, although at lower frequencies (less physiological) it was increased. Moreover, apocynin further reduced this Ca^2+^ sensitivity of aged myocytes. This suggests that apocynin induces a redox reversible modification of the myofilaments. It has been shown that an increase in *S*-nitrosylation or a reduction in *S*-glutathionylation of myofilaments regulatory proteins such as the cardiac myosin-binding protein C (cMyBP-C), troponin C, and troponin I, might induce a reduction of myofilaments sensitivity to Ca^2+^ [32,33,34]. This could be the case for the effect of apocynin in aged myocytes.

Therefore, the data suggest that one of the effects of NOX inhibitors was to improve diastolic function in aged rat hearts.

### Study Limitations

The present study has several limitations. First, the inhibitors used are not selective for the different NOX isoforms, and hence it is not possible to distinguish whether the effect observed is due to NOX2 or NOX4, which are the main isoforms present in the cardiac myocytes. Second, although it is shown that NOX inhibition reduced the oxidative stress, the exact redox modifications on target proteins such as the excitation–contraction coupling machinery or the myofilaments responsible for the effects observed were not identified.

## 4. Materials and Methods

### 4.1. Animals

According to the rat lifespan, we used adult (5–6 month-old) and aged (20–24 month-old) male and female rats [35]. All procedures were performed in conform to the NIH Guide for the Care and Use of Laboratory Animals. The protocol (code 2016-05-A, 03- 31-2016) was approved by CIECUAL of Universidad de Talca.

To study changes in left ventricular function with age, studies were performed in Langendorff isolated perfused beating hearts. Briefly, male and female rats were injected intraperitoneally with thiopental (10 mg/Kg) and subsequently with heparin (100 U/Kg). Once the animal was under deep anesthesia, (checked as the complete absence of sensitive reflexes), the thorax was rapidly opened, the heart excised, and the aortic root was cannulated to initiate retrograde perfusion. A Krebs–Henseleit buffer was used as the perfusate (in mM: NaCl, 118; KCl, 4.7; CaCl_2_, 1.75, MgSO_4_, 1.2; KH_2_PO_4_ 1.2, NaHCO_3_ 25; glucose, 11) equilibrated with 95% O_2_ and 5% CO_2_ (37 °C). A polyvinyl balloon-tipped catheter made in our laboratory from polyethylene tubing (PE-50) was inserted into the left ventricle via the mitral valve through a small opening in the left atrium. The balloon was filled with saline solution to adjust the end diastolic pressure (EDP) at 5 mmHg. This balloon was used to monitor left ventricular pressure via a pressure transductor (Ohmeda Instruments P23 XL, Madison, WI, USA) positioned at the level of the heart. A second transducer, connected to a side arm of the aortic perfusion catheter, was used to monitor coronary perfusion pressure. Hearts were paced at a rate of 300 beats/min with an electrical stimulator Grass S6 (Quince, MA, USA). Left ventricular peak systolic pressure (LVPsP), maximum rate of contraction (+dP/dt), and maximal rate of relaxation (−dP/dt) were monitored using a Powerlab System (AD Instruments, Castle Hill, Australia), used as the index of global myocardial performance. All data were continuously acquired by Lab Chart software version 7 for Windows.

### 4.2. Isolated Heart Experimental Protocols

All hearts were subjected to a stabilization period (15–20 min) before baseline measurement. After that, hearts were perfused with isoproterenol (Sigma-Aldrich, St. Louis, MO, USA) at increasing concentrations (1 nM–1 µM range). A group of aged hearts was treated with 100 μmol/L apocynin (Sigma-Aldrich, St. Louis, MO, USA), a non-selective inhibitor of NADPH oxidase, before and during the isoproterenol challenge. Another group of aged hearts was treated identically with another NADPH oxidase inhibitor, VAS2870 [36], 20 μM (Merck, Darmstadt, Germany).

### 4.3. Isolation of Rat Ventricular Myocytes

Myocytes were isolated from the left ventricle of male rats as previously described [37]. Briefly, heart were rapidly removed from an anesthetized rat and mounted onto a Langendorff system. After being perfused with a modified Krebs–Henseleit buffer (mM: NaCl, 118; KCl, 4.7; MgSO_4_, 1.2; KH_2_PO_4_ 1.2, NaHCO_3_ 25; glucose, 11; butanedione monoxide 10; taurine 5) equilibrated with 95% O_2_ and 5% CO_2_ (37 °C) for 10 min, the heart was digested for ≈75 min with collagenase type II 1 mg/mL (Worthington, NJ, USA) and protease IV 0.1 mg/mL (Sigma-Aldrich, Saint Louis, MO, USA) in the modified Krebs–Henseleit buffer. The digested heart was then removed from the cannula, and the left ventricle was cut into small pieces in the modified Krebs–Henseleit buffer. Tissue pieces were centrifuged, and the pellet of cells was resuspended in a modified Tyrode solution (Ca^2+^ free). Extracellular Ca^2+^ was added incrementally back to 1.3 mM over period of 60 min. Only rod-shaped cells with clear striation pattern without blebs or other morphological alterations were selected for mechanical and intracellular Ca^2+^ studies.

### 4.4. Measurement of Cardiomyocytes Shortening/Relengthening

Mechanical properties of cardiomyocytes were assessed using a video-based sarcomere length monitoring system (Ion Optix, Milton, MA, USA). In brief, myocytes were transferred to a Lucite chamber on the stage of an inverted microscope (Nikon TE 200, Tokyo, Japan) and superfused at 37 °C with a Tyrode solution containing 1.3 mM Ca^2^. Myocytes were field-stimulated and sarcomere length was monitored in real time using an IonOptix iCCD camera and specialized data acquisition software (IonWizard 6.3 SarcLen Acquisition System, IonOptix, Milton, MA, USA). The myocytes were field stimulated to contract at a frequency of 0.5, 1, 2, 3, and 4 Hz using a pair of platinum wires placed on opposite sides of the chamber and connected to a MyoPacer Field Stimulator (Ion Optix). The myocyte being studied was displayed on the computer monitor using an IonOptix MyoCam CCD100M camera side-mounted onto the microscope. Changes in the average sarcomere length were determined by fast Fourier-transform of the Z-line density trace to the frequency domain using the acquisition software noted above. Changes in sarcomere length during shortening and relengthening was captured and converted to digital signal. Sarcomere shortening and relengthening were assessed using the following indices: peak shortening (PS), percentage of shortening (%S = ∆*L*/*L*_0_ × 100), time to 50% peak shortening (TS_50_) and time to 50% peak relengthening (TR_50_), and Tau constant of relaxation. Twitch amplitude was computed as the difference between diastolic and peak systolic sarcomere lengths. Percentage of sarcomere shortening was expressed as the ratio of absolute twitch amplitude to diastolic sarcomere length.

### 4.5. Intracellular Calcium

Cardiomyocytes were loaded with 1 μM fura 2-AM (Thermo Fisher Scientific, Waltham, MA, USA) in the dark for 15 min, and fluorescence measurements were recorder with a dual excitation fluorescence photomultiplier system (Ionoptix). Cardiomyocytes were placed in a Lucite chamber on the stage on a Nikon Eclipse E200 inverted microscope and imaged through a Fluor 40× oil objective. Intracellular Ca^2+^ concentration ([Ca^2+^]*_i_*) was determined by exciting with light from a Xenon lamp passed alternately through 365 and 380 nm filters. The emission fluorescence was reflected through a barrier filter (510 ± 15 nm) to a photomultiplier tube. The fura-2 emission ratio at 360/380 nm represents an non-calibrated signal of [Ca^2+^]*_i_*,

#### 4.5.1. Analysis of Ca^2+^ Transient Data

This was determined as the percentage of change between the levels of [Ca^2+^]*_i_* in systole and diastole: [Ca^2+^]*_i_* = 100 *×* (*F* − *F*_0_)/*F*_0_). The time course of Ca^2+^ fluorescence signal decay (the duration at which Ca^2+^ transient decays 50%) was calculated to determine [Ca^2+^]*_i_* clearing rate. The time constant (*τ*) for myocyte relengthening and the Ca^2+^ transient decay is calculated by fitting the decay of the transients to the exponential curve: *Y*(*t*) = *a*·*e*^−^*^λt^* − *b*. Where *a* = twitch amplitude or Ca^2+^ transient; *b* = baseline value; *λ* is calculated and *τ* = 1/*λ*.

#### 4.5.2. Assessment of Calcium Myofilament Responsiveness

Changes in myofilament responsiveness to Ca^2+^ were assessed using the steady-state relation between cell length and [Ca^2+^]*_i_* in intact single cardiac myocytes tetanized by high-frequency (40 Hz) stimulation after exposure to thapsigargin (0.2 µmol/L for 15 min), as described previously [38]. Thapsigargin disables the SR and thus enables tetanization of otherwise intact myocytes. High-frequency electrical stimulation after thapsigargin treatment results in the effective summation of the repetitive transmembrane flux of Ca^2+^ current (because of the absence of sarcoplasmic reticulum Ca^2+^ sequestration and periodic release) to achieve a steady-state level of myoplasmic Ca^2+^ substantially elevated above resting levels, at the point that Ca^2+^ influx is balanced by the rate of Ca^2+^ extrusion (via Na/Ca exchanger). With this approach, the [Ca^2+^]*_i_* can be reversibly clamped near peak systolic levels during the tetanic contracture for 10 to 20 s and then rapidly returned to resting levels on cessation of electrical stimulation via normal Na/Ca exchange mechanisms. Thus, changes in the degree of cell shortening between tetani clamped at the same [Ca^2+^]*_i_* level can be attributed to changes in the relative myofilament responsiveness to Ca^2+^.

Phase loop analysis. In addition, the myofilaments dynamics was studied by plotting the levels of [Ca^2+^]*_i_* against its corresponding value for sarcomere shortening, to obtain information of the activating and the relaxation phases of the myofilaments, as has been previosly reported [39,40]. The relationship sarcomere shortening-[Ca^2+^]*_i_* relationships were determined experimentally and fitted to the Hill equation to obtain Sarc Shtering mx, or maximal [Ca^2+^]_I_ activating shortening and ECa_50_ and the Hill slope as follow.

$$Sarcomere\;Shortening=Min+\frac{\left(Max-Min\right)}{1+10^{\left(LogEC50-\left[{Ca}^{2+}\right]\right)\cdot Hill\;Slope}}$$

### 4.6. Oxidative Stress Analysis

Oxidative stress was evaluated using the levels of thiobarbituric acid reactive substances (TBARS). TBARS were quantified using heart homogenate supernatants, that were mixed with SDS (8% *w*/*v*), thiobarbituric acid (0.8% *w*/*v*), and acetic acid (20% *v*/*v*) and heated for 60 min at 90 °C. Precipitated material was removed by centrifugation, and the absorbance of the supernatant was determined at 530 nm. Levels of TBARS were calculated using a calibration curve with malondialdehyde (MDA, Sigma-Aldrich) in a Multiskan GO Microplate spectrophotometer (Thermo Scientific, Rockford, Ratastie, Finland).

#### NADPH Oxidase Activity

NADPH oxidase activity was measured indirectly as the reduction of cytochrome C by superoxide (O_2_^−^) in the presence of NADPH with an increase in absorbance at 550 nm. For this assay, heart homogenates were centrifuged at 1000 rpm for 10 min at 4 °C. After this, the supernatant was removed and centrifuged at 12,000 rpm for 10 min at 4 °C. The resulting supernatant (100 μg of protein) were mixed with working solution (300 mmol/L KH_2_PO_4_, 0.1 mmol/L EDTA, 36 μmol/L cytochrome C, pH 7.8, 20 μL of 50 mmol/L KCN, and 100 µmol/L NADPH. Absorbance measurements at 550 nm were performed during 35 min in a Multiskan GO Microplate spectrophotometer. As a positive control we used a membrane fraction of HL-60 cells (human leucoplast cell line; ATCC-NoCCL 240) differentiated to monocytes by DMSO treatment. In these conditions, HL-60 cells express NOX2.

### 4.7. Western Blotting

Tissues samples from the heart ventricle were removed and homogenized in a lysis buffer containing 50 mM Tris (pH 7.4), 30 mM NaCl, 2 mM EDTA, 0.1% SDS, and 1% protease inhibitor cocktail (Sigma-Aldrich, St. Louis, MO, USA). Samples were then centrifuged at 1500 rpm for 10 min at 4 °C. The protein concentration of the supernatant was determined by the bicinchoninic acid assay using bovine serum albumin as standard (Thermo Scientific, Rockford, IL, USA). Same amount of the protein (100 μg protein/lane) and pre stained molecular weight marker (PageRulerTM Plus Thermo Scientific, Rockford, IL, USA) were separated on 7.5 and 12% SDS-polyacrylamide gel, then were transferred to nitrocellulose membranes (0.2 μm pore Bio-Rad Laboratories, Hercules, CA, USA). Membranes were incubated for 1 h in a blocking solution containing 5% albumin in TBT-T buffer, and were then incubated with anti-SERCA2a (Santa Cruz Biotechnologies, Santa Cruz, C, 1:1000), anti-PLB (Badrilla, Leeds, UK, 1:1000), anti NOX2 (BD Biosciences, Franklin Lakes, NJ, US,), anti-NOX3 (Santa Cruz Biotechnology), anti-NOX4 (Thermo Scientific), anti-phospho-phospholamban Ser16/Thr17 (Cell Signaling Technology, Danvers, MA, USA) 1:1000, and anti-GADPH (Santa Cruz) antibodies at 4 °C overnight all at a dilution of 1:1000. After washing blots to remove excess primary antibody binding, blots were incubated for 1 h with horseradish peroxidase (HRP)-conjugated secondary antibody (Santa Cruz Biotechnologies, 1:2000). Antibody binding was detected using enhanced chemiluminescence reagent (SuperSignal^®^ West Femto Maximum Sensitivity Substrate, Thermo Scientific, Rockford, IL, USA) and film were digitalized and scanned and the intensity of immunoblot bands detected with Image J software version 1.49v (Wayne Rasband, National Institute of Health, Bethesda, MD, USA).

### 4.8. Statistical Analysis

Sample size (animals) was estimated by performing power analysis based on previous data for contractility parameters, to achieve an 80% power (20% β-error) with α-error <5%. Results are expressed as mean ± standard deviation or mean ± standard error where indicated. Comparison between two groups were made using the unpaired, two tails *t* student test. Analysis of concentration-response curves and shortening-frequency response experiments were performed using two-way analysis of variance (ANOVA) with Bonferroni as post hoc analysis and one way analysis with Neuman–Keuls post-hoc analysis, where appropriate. Isoproterenol concentration–response curves were fitted to the Hill equation as described above. For non-parametric data, Kruskal–Wallis test was applied, with Dunn’s multiple comparisons test as post-hoc analysis. Statistical significance was set at a value of *p* < 0.05. These analyses were performed using the GraphPad Prism 5 software (San Diego, CA, USA).

## 5. Conclusions

Aged rat hearts presented a diminished response to isoproterenol, which was restored upon pharmacological NOX inhibition. In aged myocytes, twitch contractions, presented slowed kinetics, and reduced myofilaments Ca^2+^-sensitivity. NOX inhibition increased [Ca^2+^]*_i_* transients amplitude and improved Ca^2+^ kinetics. Aged rat hearts presented lower levels of SERCA2 and reduced phospholamban phosphorylation upon adrenergic stimulation that was reversed by NOX inhibition.

Understanding how NOX activity influences the aged myocardium function at the cellular level may help to develop strategies for the prevention and treatment of cardiovascular disease in this population. Pharmacological targeting of NOX in aging may help to improve cardiac function.

## Figures and Tables

**Table 1:** Baseline characteristics of isolated hearts. Mean ± standard deviation.

	Adults	Aged	*p*-Value
*N* (hearts)	12	19	-
Age (months, range)	5–6	20–24	-
Heart weight (g)	1.45 ± 0.08	2.19 ± 0.13	0.00069551
Body weight (g)	344.4 ± 50	702.5 ± 82	<0.0001
Developed pressure (mmHg)	108.3 ± 53.5	41.4 ± 19.1	0.02956983
dP/dt_max_ (mmHg/s)	2808 ± 1269	1170 ± 815	0.0281473
dP/dt_min_ (−mmHg/s)	−2054 ± 988	−743 ± 650	0.02598013
Coronary perfusion pressure (mmHg)	60.5 ± 34	37.9 ± 9.9	0.17102549

**Table 2:** Baseline values for sarcomere shortening and intracellular calcium of isolated myocytes (mean ± standard deviation).

Sarcomere Shortening	Adults	Aged	*p*-Value
*N* (hearts)	5	4	-
*N* (cells)	17	22	-
Sarcomere Length (µm)	1.74 ± 0.066	1.73 ± 0.077	0.289
Contraction Amplitude (µm)	0.189 ± 0.056	0.17 ± 0.07	0.185
% Peak to baseline	10.8 ± 3.1	9.76 ± 4.1	0.185
Time to peak 50% (s)	0.072 ± 0.013	0.107 ± 0.04	<0.005
Time to 50% baseline (s)	0.379 ± 0.12	0.542 ± 0.09	<0.005
Tau relaxation (s)	0.193 ± 0.407	0.186 ± 0.08	0.407
**Intracellular Ca^2+^**			
Diastolic [Ca^2+^]*_i_* (fura ratio)	1.2 ± 0.18	1.06 ± 0.05	0.0051
Systolic [Ca^2+^]*_i_* (fura ratio)	0.20 ± 0.12	0.27 ± 0.2	0.0916
% Peak to baseline	17.1 ± 11.1	26.7 ± 20.7	0.044
Time to peak 50% [Ca^2+^]*_i_* (s)	0.04 ± 0.005	0.05 ± 0.002	0.0067
Time to 50% baseline [Ca^2+^]*_i_* (s)	0.25 ± 0.08	0.35 ± 0.01	<0.005
Tau [Ca^2+^]*_i_* decay (s)	0.26 ± 0.148	0.25 ± 0.06	0.420

## Supplementary Materials

Supplementary materials can be found at http://www.mdpi.com/1422-0067/19/8/2404/s1.
