# Supplementary material for: NOX Inhibition Improves β-Adrenergic Stimulated Contractility and Intracellular Calcium Handling in the Aged Rat Heart
Source: Int J Mol Sci. 2018 Aug 15;19(8):2404. doi: 10.3390/ijms19082404 (PMC6121436; doi:10.3390/ijms19082404)
Supplement: Supplementary file 1 [file ijms-19-02404-s001.pdf]

Supplementary Figure 1

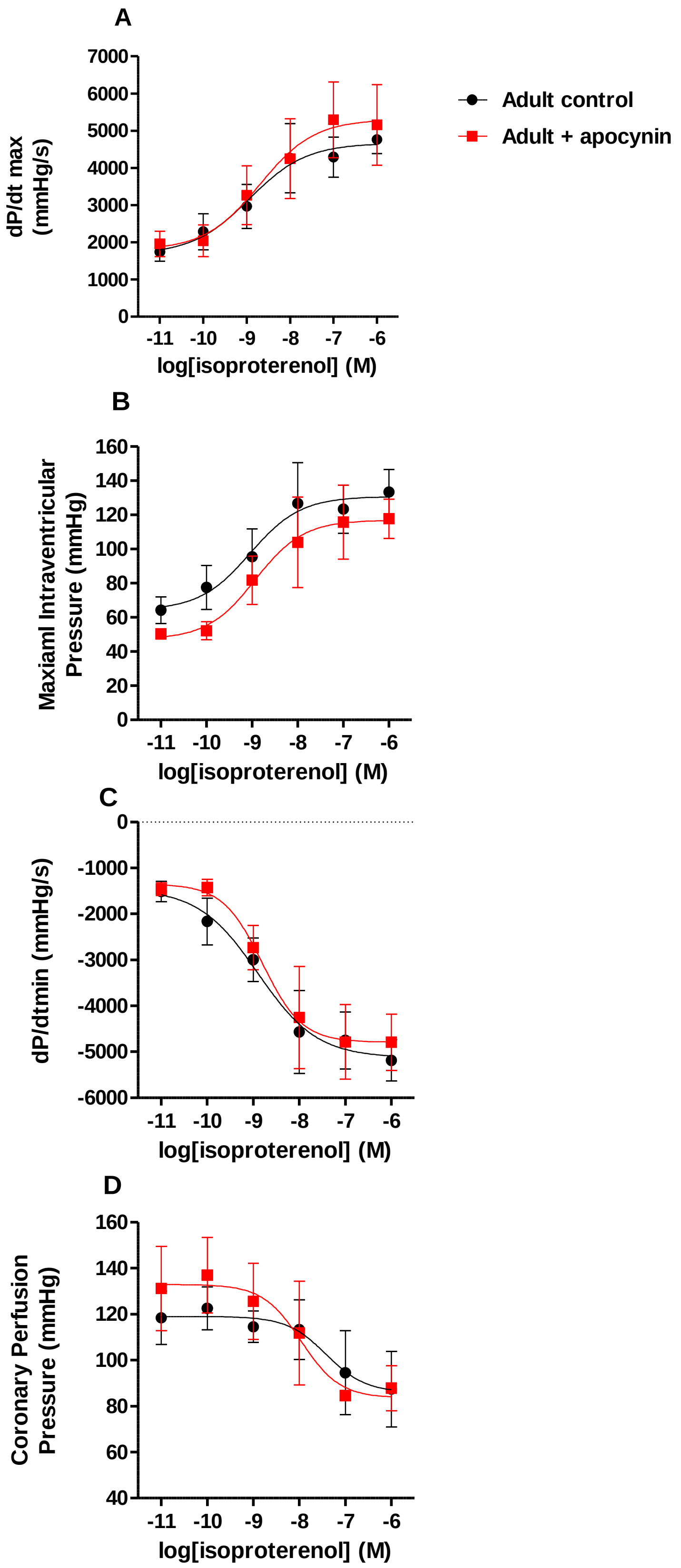

Supplementary Figure 1

Supplementary figure 1. Hemodymanics response of adult isolated hearts treated with (n= 4) or without (n=6) with apocynin 100 µM. A, ionotropic response, evaluated as dp/dt_{max}. B, developed intraventricular pressure. C Ventricular relaxation, evaluated as $dp/dt_{min.}$ D, coronary prefusion pressure.
